# Nature Elements and Fundamental Motor Skill Development Opportunities at Five Elementary School Districts in British Columbia

**DOI:** 10.3390/ijerph14101279

**Published:** 2017-10-24

**Authors:** Christopher Lim, Andrew M. Donovan, Nevin J. Harper, Patti-Jean Naylor

**Affiliations:** 1 School of Exercise Science, Physical and Health Education, University of Victoria, P.O. Box 1700 STN CSC, Victoria, BC V8W 2Y2, Canada; chrislim@uvic.ca (C.L.); andrewdo@uvic.ca (A.M.D.); 2 School of Child & Youth Care, Human and Social Development, University of Victoria, Victoria, BC V8W 2Y2, Canada; njharper@uvic.ca

**Keywords:** naturescape, children, school, playground, physical literacy, functional motor skills, nature

## Abstract

The majority of Canadian children are not physically active enough for healthy development. School playgrounds are a primary location to promote physical activity and motor skill practice. The benefits of children’s play in nature have also been highlighted, but few studies have evaluated children’s access and exposure to nature for play on school grounds. This study examined children’s access to nature on school grounds and the opportunities afforded by those natural elements for motor skill practice. Results: Extensive naturescapes (multiple nature elements in one setting) were not common, and natural elements were limited, ranging from 1.97 to 5.71 elements/school. The most common element was a forested area (26.5% of all natural elements identified). In comparison to built structures, the number of natural elements was low. Some elements differed between school districts and appeared to be related to local geography and terrain (hilly, rocky terrain, tidal flats, etc.). Our assessment showed that naturescape elements afforded opportunities for the development of some key fundamental motor skills (FMS), specifically, locomotor and stability skills, but opportunities to develop manipulative skills were limited. To maximize potential FMS development, physical literacy, and psycho-social benefits, additional elements or more comprehensive multi-element naturescapes and facilitation (social or environmental) are recommended.

## 1. Introduction

Canadian children are currently not physically active enough for healthy development; with only 9% of boys and girls meeting recommended levels of physical activity (PA) [1]. With an emerging obesity epidemic, many different approaches to the promotion of PA across a variety of settings where children spend time are needed [1,2]. Models such as the social ecological model explain the effects of environmental inputs on health behavior [3,4,5,6]. Studies have shown for instance that macro environment characteristics affect PA levels among residents of a neighborhood [7].

Natural spaces and features are key components of any environment. Current trends of urbanization, dwindling natural spaces, and children’s reduced exposure to nature have led to research about the effects of nature on both physical and mental health and wellbeing [8,9]. Access to green spaces and nature may have a greater impact on youth populations, especially in regard to development [10]. A 2014 review of literature by Hartig et al. [9] indicated that exposure to nature could contribute to physical benefits, buffer symptoms of obesity and diabetes, and benefit children’s overall development. Norwegian children who consistently played in nature during recess performed better in motor skills tests than children who played on traditional playgrounds [11] and showed improved motor fitness [12]. Additionally, experiences in natural environments have enhanced children’s attitudes toward increased PA [13,14], positively affected children’s social, emotional and cognitive development [15], and reduced student stress [16]. What is clear in the literature is that natural play elements which engage children in active and dynamic play produce a wide array of physical, social, and emotional benefits [17,18]. 

Although there is a great deal of research about macro environments, we hoped to build on social ecological assessments of the microenvironment, such as that by Gubbels et al. [19] who found that interactions between microsystem elements in part determined a child’s PA. Maas et al. [20] found the amount of green space within a one-kilometer radius of an individual’s residence significantly affected one’s perceived health. This relationship being especially strong among the elderly and youth in urban areas [20]. Additionally, altering the playground microenvironment in terms of playground markings or structures such as seating has been shown to alter children’s PA time and intensity [21,22,23]. 

Naturescapes are design elements attempting to bridge the gap between modern playscapes and wilderness or more natural physical areas [24]. This may take the form of increased variability in topography, forested areas, boulders, logs or gardens. The literature reviewed suggests natural elements added to school grounds act as a catalyst for child development and wellbeing through increased affordances for creative and ‘risky play’ [14,18]. The potential contribution of natural elements as specific features of a playground environment to the development of gross motor movement skills remains unexamined. Within this context we posed the primary research questions: (1) what nature elements are currently available for child play on school grounds? and (2), what is the potential of these school playground microenvironment features to provide motor skill opportunities for elementary school-aged children? We provide context for the analysis using community-level socio-demographic and vulnerability measures from existing population health data sets and school district information. A secondary analysis explored if there were regional differences in naturescape access and socio-demographic variables.

## 2. Experimental Section

### 2.1. Sample Selection

In total, 99 primary schools in five British Columbia districts (Greater Victoria, Saanich, Sooke, Richmond, and Gulf Island) were audited. Districts were selected for geographic proximity to our research team and variation in the geographic (physical environment, landscape) and demographic (socioeconomic status (SES), urbanization) context. Our study includes schools from small, medium, and large urban population communities as defined by Statistics Canada. Some of the schools were more rural. As observations were conducted in during the summer months (in publically accessible spaces) and human participants were not involved, the Human Research Ethics Board at the University of Victoria waived the need for ethical approval.

### 2.2. School and School District Context

Demographic and socioeconomic data was obtained from existing population data sets for the region or community where each school was located. The Early Development Instrument 2011–2013 Wave 5 data (EDI) [25] as used to describe population-based vulnerability rates by school geographical area. EDI is a school readiness instrument completed by kindergarten teachers upon school entry that identifies vulnerability overall and on six domain sub-scales (physical and mental well-being, social competence, emotional maturity, language and cognitive development, and communication skills and general knowledge). Scores represent the percentage of the population that is vulnerable and a lower percentage indicates lower vulnerability. BC Statistics [26] and Statistics Canada 2016 census data [27] were used to describe median family income and community size. School population size was taken from district information and the total play space available at the school was gathered by the research team using a measuring wheel.

### 2.3. Categorization and Availability of Natural Elements

A naturescape was defined as the presence of at least one of the following predetermined elements (forested area, boulders/logs, elevated area, trails, garden area, natural playground, other). Each element category was defined during the pilot stage and prior to study implementation. These elements were defined and incorporated into a playground checklist (see Table 1) and pilot tested. Inter-rater reliability for the checklist was 96.9%. However, two members of the research team went to every school ground and tallied the total number of each element present (frequency) using the playground element checklist. Any issues with categorization were addressed through negotiated consensus.

### 2.4. Fundamental Motor Skill Opportunities (FMS)

Potential FMS opportunities for each natural element were agreed upon through negotiated consensus among the researchers. It should be noted that the opportunities were assessed from an adult perspective. FMS opportunities were categorized as being related to either (1) *locomotor skills*; (2) *manipulative skills*; or (3) *stability skills* and all potential skills were counted for each element (e.g., a climbing structure would provide a count for both locomotor and stability). We used a set list of FMS skills (*n* = 27) taken from previous literature [28]. Locomotor skills included: climbing, galloping, hopping, jumping, leaping, running, skipping, sliding, and walking. Manipulative skills included: catching, dribbling, kicking, punting, striking, and throwing. Stability skills included: balancing, bending, body rolling, dodging, dynamic balancing, inverted supports, landing/stopping, pivoting, stretching, swinging, turning, and twisting.

### 2.5. Analysis

Data was entered into Microsoft^®^ Excel for Windows v. 14.16.9 (Microsoft, Redmond, WA, USA) and analyzed using IBM SPSS Statistics for Windows Version 20.0 (IBM, Armonk, NY, USA). Descriptive statistics for each District and across Districts were calculated for number of nature elements and potential motor skill development opportunities. To explore the impact of context on naturescape access, descriptive statistics were also generated and one-way analysis of variance was used to determine differences in access to nature elements and demographics across districts. 

## 3. Results

### 3.1. Demographics

Table 2 provides the demographic details for the communities where schools were observed, including average annual income, child vulnerability (overall and physical health and well-being sub-scale), community size and type. A majority of the schools were in an urban/suburban settings. At the level of demographic and vulnerability detail available, most communities (*n* = 10) were classified as small population centers, with two classified as medium and two large urban centers. It should be noted that Greater Victoria, a large urban population center, is broken into 12 municipalities, but observable differentiation is challenging without maps and signage. Statistics Canada has recognized that at the population level, rural to urban areas exist on a continuum. Some of the schools in this study exist within small urban centers but are more rural in their location. A reliable source of classification was not available and we did not develop our own.

### 3.2. Access to Naturescape Elements

The number, mean, and range of elements across districts are displayed in Table 3. There was access to all natural elements within each district. The average number of nature elements was 3.7 (SD 3.99; range 0–20 elements) per school ground, whereas the average number of man-built playground structure elements (e.g., balancing beam, monkey bars, island hoppers) for example was 58.7 (SD 25.7; range 0–139 elements) per playground or for court elements (e.g., basketball hoops, nets etc.) this value was 18.4 (SD 7.0; range 4–47) (data not shown). The frequency in which the elements appeared varied with forest elements appearing the most, followed by garden areas and boulder and log elements, while ‘natural playgrounds’ appeared the least throughout all districts. However, as indicated by the standard deviation and ranges the variability from school to school was large.

### 3.3. Comparing Demographics and Nature Elements across Districts

There were significant differences in socioeconomic status (median family income), vulnerability and play space measures as well as the number of nature elements across the districts. Table 4 provides the main effects from the ANOVA and the results of the post hoc tests to determine where the differences were. Although socio-economic and vulnerability levels differed significantly across the districts, this did not appear to follow the pattern of the nature element access differences. Measures of socioeconomic status and EDI were not significantly correlated with nature element access with the exception of median family income and garden areas (*r* = −0.284, *p* < 0.004; data not shown). When comparing district access to naturescapes there were some variations. For instance, Greater Victoria elementary schools had significantly more access than Richmond schools to boulders and logs, elevated areas, trails, and the overall total number of nature elements on each school playground. There was also more garden area access in Gulf Island elementary schools as compared to Sooke elementary schools.

### 3.4. FMS Opportunities

Table 5 shows there was potential for the development of 16 of 27 possible FMS based on the nature elements observed. While opportunities for the development of stability and locomotor skills appeared common for nature elements, opportunities for the use of manipulative skills were not. The FMS development opportunities most afforded by natural elements were: balancing, dynamic balance, bending, walking, running, climbing, and jumping. No opportunities for manipulative skill practice were identified.

## 4. Discussion

Prior to this study we found no literature on availability of natural elements at school grounds in British Columbia. Nor did we find information about the impact of playing in natural environments on gross motor skill development. With a growing literature highlighting the short [29,30,31] and longer-term importance of motor skill development [32] and a child’s micro environment to PA (particularly school grounds) [33,34,35], as well as the importance of exposure to nature to mental and physical well-being [36], we felt it was important to determine whether children were being exposed to natural features at their elementary schools and to assess whether these natural features afforded opportunities for motor skill development. 

The data collected supports the idea that children could gain motor skill practice through naturescape accessibility on school grounds [14,37,38], albeit primarily locomotor and stability skill development. This study highlights naturescapes as an area to further explore PA, FMS, and physical literacy intervention opportunities on school grounds. Further, the differences in the broader context within which schools exist, some of which were not identified in our data collection (e.g., topography, socio-economic status, child vulnerability, school size, social mileu), draw attention to the considerations related to providing access and types of nature elements and ultimately PA experiences.

Although we did not set out to assess opportunities for risky play, the natural elements observed in this study aligned well as affordances for outdoor risky play such as climbing to height and gaining speed running downhill from elevated areas [39]. These ‘risks’ are mostly perceived by children as ‘risky’ and are suggested to assist children in their development through subsequent reduction of anxiety or phobias in later life related to height and speed as in the previous examples.

Our findings should be viewed in light of study strengths and limitations. The audit processes were pilot tested and inter-rater reliability was strong across researchers and over time within the study. We explored the literature and created definitions for nature elements and opportunities for motor skills through observation and a negotiated consensus process among our research team. It is possible that our assessment, although consistent, did not accurately represent the actual motor skill opportunities as the observations were conducted from the perspective of adults and children were not directly observed. Additionally, we did not account for the total size of each element which would affect the actual availability to children. Therefore, future research in this area should examine how children engage with natural elements, the actual motor skills used in nature spaces, and any limitations to accessibility for children that may be governed by the size of naturescape elements, policies, and supervision at the school. For instance, we heard anecdotally that one school had a rule forbidding the children to use of the forested area for play.

A positive relationship between amount of contact with nature and health has been suggested [40] and our study highlighted school grounds as one setting for further inquiry in this regard. Our descriptive study provides no indication of whether the existing frequency and type of nature elements is adequate however. The broad overview of natural elements provides a foundation for school ground and playground design decisions, where affordable and accessible natural elements may be added to promote healthy child development and well-being (e.g., increased topographical variability such as hills and logs). School system decision-makers need to be aware of the limitations in terms of manipulative skill development and consider existing topography and socio-economic factors as they decide where to invest in ‘playground naturalization’ efforts.

## 5. Conclusions

School ground naturescapes were not common and where plentiful, were often naturally occurring features of the area’s topography. The naturescapes visited during this study provided a clear opportunity for PA, the development of many key FMS, and likely increased creative and risky play. Purposeful facilitation of manipulative skill development appears necessary. Natural play areas, as with other types of play environments, could benefit from additional structure and equipment in order to best maximize FMS development, even if those “structures” include trees, boulders, and other climbable natural elements. It is our hope that this study and subsequent research on PA, FMS, and physical literacy be used thoughtfully by key community decision-makers such as city planners and school officials.

## Figures and Tables

**Table 1:** Operational definitions for naturescape elements and the associated potential fundamental movement skill (FMS) development opportunities.

Nature Scape	Definition	Potential FMS Opportunities for Stability, Locomotor, and Manipulative Skill Development		
Forested Area	An area comprising multiple trees, bushes, stumps, and/or other plants; not property border trees that are used to ‘fence’ or identify school yard boundaries or separate the school grounds from the neighborhood.	Stability	Locomotor	Manipulative
		Twisting	Climbing	not applicable
		Turning	Running	
		Balance	Walking	
		Dynamic Balance		
		Bending		
Natural Playground	A built structure primarily using natural elements to represent a nature space. For example, log bridges and stump island hoppers.	Stability	Locomotor	Manipulative
		Balancing	Climbing	not applicable
		Bending	Hopping	
		Dynamic Balance	Jumping	
		Landing	Leaping	
		Twisting	Running	
		Turning	Walking	
Boulder/Logs	The presence of a ‘set’ of boulders and logs that have an unstructured layout and are at least large enough to stand on and/or climb on	Stability	Locomotor	Manipulative
		Balance	Climbing	not applicable
		Bending	Jumping	
		Dynamic Balance	Walking	
			Running	
Elevated Area	An unstructured hill area or mounds (dirt, grass, and/or rocky ground) that children can climb up or down on	Stability	Locomotor	Manipulative
		Body Rolling	Running	not applicable
		Balance	Walking	
		Bending		
		Dynamic Balance		
Garden Area	A controlled area that is dedicated to growing plants (either flowers and herbs or vegetables)	Stability	Locomotor	Manipulative
		Bending	Not applicable	not applicable
		Stretching		
		Balance		
Trails	A structured or semi-structured pathway around natural elements (typically they have chips, crushed rocks, and/or small wooden borders)	Stability	Locomotor	Manipulative
		Balance	Galloping	not applicable
		Dynamic Balance	Hopping	
			Leaping	
			Running	
			Skipping	
			Sliding	
			Walking	

**Table 2:** Demographics for school neighbourhood/municipality including indicators of socio-economic status, vulnerability (median income, vulnerability assessed by the Early Development Instrument Wave 5—EDI), population, and community type.

School District	EDI Neighborhood/s	No. of Schools/EDI Neighbourhood	Vulnerability EDI Score—% Vulnerable on More Than One Sub-Scale/100	Median Family Income in CAD Dollars	Population of the Municipality/Region Schools Are In	Type ^1^
Greater Victoria	Oak Bay—Fairfield	3	0.18	101,531	18,094	SP
	Hillside-Fernwood	3	0.3	66,135	85,792 *	MP
	Cedar Hill—Mt Tolmie	2	0.18	82,373	114,148 *	LUP *
	University Gordon Head	3	0.28	89,627	114,148 *	LUP *
	High Quadra	4	0.2	51,935	114,148 *	LUP *
	Burnside—Mayfair	2	0.42	66,018	85,792 *	MP *
	Downtown—James Bay	2	0.29	64,880	85,792 *	MP *
	Esquimalt—Vic West	2	0.27	72,368	17,655	SP
	Carey—Glanford Strawberry Vale	5	0.3	82,599	114,148 *	LUP *
	View Royal Thetis Lake	2	0.32	81,422	10,408	SP
Gulf Islands	Gulf Islands	8	0.24	59,891	10,577	SP
Saanich	Cordova Bay	2	0.17	99,331	114,148 *	LUP *
	Sidney	1	0.29	72,392	11,672	SP
	Deep Cove	2	0.16	97,254	11,249	SP
	Central Saanich	3	0.23	88,081	16,814	SP
Sooke	Highlands	2	0.31	95,869	2225	DP
	Langford	4	0.36	78,148	35,342	MP*
	Colwood—Royal Roads	5	0.31	91,245	17,655	SP
	Metchosin	2	0.23	88,674	4708	SP
	Sooke West Coast	4	0.33	76,323	13,001	SP
Richmond	City Centre North	2	0.43	50,910	198,309 *	LUP *
	Shellmont	3	0.35	71,879	198,309 *	LUP *
	City Centre South	3	0.4	52,820	198,309 *	LUP *
	Broadmoor	8	0.36	71,254	198,309 *	LUP *
	Bridgeport-East Cambie	3	0.29	71,704	198,309 *	LUP *
	Hamilton	1	0.36	94,010	198,309 *	LUP *
	Blundell	3	0.42	72,364	198,309 *	LUP *
	Thompson-Sea Island	5	0.28	71,586	198,309 *	LUP *
	Steveston	6	0.25	94,062	198,309 *	LUP *
	Sefair	4	0.32	80,713	198,209 *	LUP

^1^ SP = small population (1000 and 29,999); MP = medium population (30,000 and 99,999), LUP = large urban population (100,000 and over) as defined by Statistics Canada [27] Note: The Greater Victoria area has 13 cities and/or towns, districts or municipal districts. * The school neighbourhood is part of a larger municipality and this number/community type represents the larger municipality.

**Table 3:** Descriptive statistics for naturescape elements across school districts.

Naturescape Elements	*n*	Mean (S.D.)	Range	Frequency
Total nature elements observed	366	3.70 (3.99)	0–20	N/A
Forest elements	97	0.98 (1.27)	0–6	26.5%
Garden areas	75	0.76 (0.32)	0–5	20.49%
Boulders + logs	72	0.72 (1.12)	0–6	19.67%
Elevated areas	66	0.67 (1.01)	0–4	18.03%
Trails	45	0.45 (0.90)	0–4	12.3%
Natural playgrounds	11	0.11 (0.82)	0–1	3.01%

**Table 4:** Analysis of Variance (ANOVA) main effects and post hoc test results examining differences in demographics and nature elements between school districts.

Variable (ANOVA Main Effect and Significance)	District	Direction of Difference	District	*p* Value
Socio-economic status F = 14.28, *p* < 0.0001	Greater Victoria	>	Gulf Islands	*p* = 0.026
	Saanich		Greater Victoria	*p* = 0.040
		>	Gulf Islands	*p* = 0.000
			Richmond	*p* = 0.011
	Sooke	>	Gulf Islands	*p* = 0.000
Early Development Index ^a^ F = 9.67, *p* < 0.0001	Richmond		Greater Victoria	*p* = 0.001
		>	Gulf Islands	*p* = 0.001
			Saanich	*p* = 0.001
	Sooke	>	Saanich	*p* = 0.001
			Gulf Islands	*p* = 0.035
School play space F = 4.18, *p* = 0.004	Richmond	>	Greater Victoria	*p* = 0.048
Number of nature areas F = 2.99, *p* = 0.002	*		*	*
Forested areas F = 2.39, *p* = 0.056	*		*	*
Natural playgrounds F = 2.34, *p* = 0.06	*		*	*
Boulder and logs F = 6.18, *p* < 0.0001	Greater Victoria	>	Richmond	*p* = 0.001
Elevated areas F = 3.76, *p* = 0.007	Greater Victoria	>	Richmond	*p* = 0.009
Garden areas F = 4.22, *p* = 0.003	Gulf Islands	>	Sooke	*p* = 0.005
Trails F = 4.66, *p* = 0.002	Greater Victoria	>	Richmond	*p* = 0.006
Total number of nature elements F = 4.99, *p* = 0.002	Greater Victoria	>	Richmond	*p* = 0.004

* No significant main effect across districts for these variables. ^a^ Greater equals more vulnerability.

**Table 5:** Number and percentage of opportunities for the development of locomotor, stability and manipulative movement skills (*n* = 27) based on the naturescape elements observed.

FMS Movement	Locomotor Opportunities		FMS Movement	Stability Opportunities		FMS Movement	Manipulative Opportunities	
	Opportunities	%		Opportunities	%		Opportunities	%
Climbing	221	22.39	Balancing	329	27.62	Catching	0	0
Galloping	44	4.46	Bending	285	23.93	Dribbling	0	0
Hopping	52	5.27	Body rolling	59	4.95	Kicking	0	0
Jumping	134	13.58	Dodging	0	0	Punting	0	0
Leaping	52	5.27	Dynamic balance	264	22.17	Striking	0	0
Running	198	20.06	Inverted supports	0	0	Throwing	0	0
Skipping	44	4.46	Landing/stopping	0	0	-	-	-
Sliding	44	4.46	Pivoting	0	0	-	-	-
Walking	198	20.06	Stretching	64	5.37	-	-	-
-	-	-	Swinging	0	0	-	-	-
-	-	-	Turning	95	7.98	-	-	-
-	-	-	Twisting	95	7.98	-	-	-
Total	987	100		1191	100		0	-
